# Concentration-dependent effects of immunomodulatory cocktails on the generation of leukemia-derived dendritic cells, DC_leu_ mediated T-cell activation and on-target/off-tumor toxicity

**DOI:** 10.3389/fimmu.2024.1527961

**Published:** 2025-01-30

**Authors:** Hazal Aslan Rejeski, Anne Hartz, Elias Rackl, Lin Li, Christoph Schwepcke, Kai Rejeski, Christoph Schmid, Andreas Rank, Jörg Schmohl, Doris Kraemer, Peter Bojko, Helga Maria Schmetzer

**Affiliations:** ^1^ Department of Medicine III, LMU University Hospital, LMU Munich, Munich, Germany; ^2^ Bavarian Cancer Research Center (BZKF), Munich Site, Munich, Germany; ^3^ Department of Hematology and Oncology, University Hospital of Augsburg, Augsburg, Germany; ^4^ Department of Hematology and Oncology, Diakonieklinikum Stuttgart, Stuttgart, Germany; ^5^ Department of Hematology and Oncology, St.-Josefs-Hospital, Hagen, Germany; ^6^ Department of Hematology and Oncology, Rotkreuzklinikum Munich, Munich, Germany

**Keywords:** blast modulation, dendritic cells, leukemia-derived dendritic cells, acute myeloid leukemia, PGE_1_, PGE_2_, OK-432, immunotherapy

## Abstract

Acute myeloid leukemia (AML) remains a devastating diagnosis in clear need of therapeutic advances. Both targeted dendritic cells (DC) and particularly leukemia-derived dendritic cells (DC_leu_) can exert potent anti-leukemic activity. By converting AML blasts into immune activating and leukemia-antigen presenting cells, DC/DC_leu_-generating protocols can induce immune responses against AML blasts. Such protocols combine approved response modifiers (i.e., GM-CSF and PGE_1_/OK-432/PGE_2_) that synergistically improve the conversion of AML blasts into (mature) DC/DC_leu_. To guide potential clinical application of these response modifiers, we analyzed three different DC-generating protocols that combine a constant GM-CSF dose with varying concentrations of PGE_1_ (Kit-M), OK-432 (Kit-I), and PGE_2_ (Kit-K). Here, we specifically aimed to assess how different response modifier concentrations impact DC/DC_leu_ generation, immune cell activation and leukemic blast lysis. We found that all immunomodulatory kits were effective in generating mature and leukemia-derived DCs from healthy and leukemic whole blood. For Kit-M, we noted optimal generation of DC-subsets at intermediary concentration ranges of PGE_1_ (0.25-4.0 µg/mL), which facilitated upregulation of activated and memory T-cells upon mixed lymphocyte culture, and efficient anti-leukemic activity in cytotoxicity assays. For Kit-I, we observed DC/DC_leu_ generation and enhanced T- and immune cell activation across a broader range of OK-432 concentrations (5-40 µg/mL), which also facilitated improved leukemic blast killing. In conclusion, our results highlight that Kit-mediated DC/DC_leu_ generation, immune cell activation and blast lysis are dependent on the concentration of response modifiers, which will guide future clinical development. Overall, DC_leu_-based immunotherapy represents a promising treatment strategy for AML patients.

## Introduction

Acute myeloid leukemia (AML) is a heterogeneous hematologic malignancy characterized by the uncontrolled proliferation of abnormally differentiated and long-lived myeloid precursors in the bone marrow and blood. Intensive chemotherapy in combination with allogeneic hematopoietic cell transplantation (HCT) can induce long-term remissions in only around 50% of AML patients and post-HCT relapse remains common (1–3). Therefore, there is a pressing need to develop novel maintenance therapies that stabilize remission.

Dendritic cell (DC)-based immunotherapy, which is either manufactured *ex vivo* and adoptively transferred or induced *in vivo*, is currently being explored as a potentially promising therapeutic option for AML (4–7). DCs are potent and multifaceted antigen presenting cells (APCs) which serve as a critical link between the innate and adaptive immune system. As sentinels of the immune system, DCs play an essential role in mediating efficient immune cell priming and stimulate leukemia specific innate and adoptive immune cells, thereby addressing blasts and installing memory cells (8–11). Moreover, DCs possess the unique ability to sense the surrounding microenvironment and initiate protective pro-inflammatory as well as tolerogenic immune responses (12). Considering the capacity of DCs to target a variety of antigens and especially by inducing memory cells, they possess the distinct ability to directly stimulate diverse immune cell subsets in a leukemia-specific manner in whole blood (WB). DC-mediated strategies could thus serve as potent maintenance therapies, since they have the ability to eradicate minimal residual disease (MRD) (13).

Various auspicious DC generation methods have been developed that can overcome the lack of immunogenicity of AML cells. DCs can be propagated from monocytes *in vitro* (moDC) (14), pulsed with leukemic peptides (15), apoptotic leukemic cells or leukemic cell lysates (16), fused with leukemic blasts (17), or electroporated with messenger ribonucleic acid (mRNA) encoding leukemia-associated-antigens (LAA) and then prepared for injection as a vaccine (16). In our previous studies, we successfully generated DCs in near physiological conditions *ex vivo* using heparinized WB or whole bone marrow, containing patients’ (potentially immune activating or inhibiting) cellular or soluble factors under physiological hypoxia or normoxia (10, 18).

Leukemia-derived dendritic cells (DC_leu_) are characterized by the expression of costimulatory dendritic antigens and the patients’ individual leukemia-specific antigens. Standard generation of DC/DC_leu_ is known to be possible with immunomodulatory Kits from leukemic or healthy WB without induction of blast proliferation (10, 19, 20). Such kits are composed of single drugs that have been approved for clinical use in patients with non-leukemic disease indications. For example, Prostaglandin E1 (PGE_1_) analogs like misoprostol or alprostadil serve mainly as vasodilators and smooth muscle relaxants across several clinical conditions such as labor induction and maintaining the patency of the ductus arteriosus in neonates (21–23). A further example is OK-432 (or Picibanil) which is a lyophilized mixture of a low-virulence strain (Su) of group A *streptococcus pyogenes* incubated with the antibiotic benzylpenicillin (24). This potent immunostimulant has been utilized as a primary therapy in the treatment of lymphangiomas (25). With respect to any potential clinical application of these drug combinations in leukemia patients, optimal concentrations need to be identified.

Here, we aimed to refine the optimal concentration ranges of three different blast modulating Kits (Kit-M, -I, -K) required to generate sufficiently high frequencies of mature DC/DC_leu_ directly from healthy or leukemic whole blood (WB) *ex vivo*. Moreover, the impact of these three different Kit-treated (DC/DC_leu_ containing) WB samples on the mediation of immune (T-cell) activation, provision of (leukemia-specific) memory cells, anti-leukemic functionality and off-target cell toxicity were analyzed. This constitutes an important and directive step for translating DC/DC_leu_-based immunotherapy into clinical application.

## Materials and methods

### Patient characteristics, sample collection and preparation

This study was carried out in accordance with the Helsinki protocol and the local Ethic Committee (VoteNo. #33905). Written informed consent was obtained from all patients. Peripheral blood was collected from AML patients (n=22) and from healthy volunteers (n=9) across multiple institutions (LMU University Hospital, Rotkreuzklinikum Munich, Augsburg, Oldenburg, Stuttgart). A detailed overview of patient features is provided in **Table 1**.

**Table 1:** Patient characteristics.

Patient characteristics							
#	Age, Sex	Disease Status	Subtype/FAB	Blast Phenotype (CD)	PB Blasts (IC)	ELN 2017 *	Performed Experiments/Kits**
**1447**	21, M	First diagnosis	pAML/ M5	**15**, 33, 34, **56**	33%	Intermediate	**DCC:** Kit-M, -I, -K **MLC:** Kit-M, -I, -K **CTX:** Kit-M, -I
**1452**	44, M	First diagnosis	pAML	13, 33, **34**, **117**	14%	intermediate	**DCC:** Kit-M, -I, -K **MLC:** Kit-M **CTX:** Kit-M
**1453**	54, F	First diagnosis	pAML/ M4	33, 64, 14, **15**, **56**	52%	adverse	**DCC:** Kit-M, -I, -K **MLC:** Kit-M, -I, -K **CTX:** Kit-M, -I, -K
**1454**	60, F	First diagnosis	sAML	13, **15**, 33, **34**, 117	33%	intermediate	**DCC:** Kit-M, -I, -K **MLC:** Kit-M, -I, -K **CTX:** Kit-M, -I
**1459**	54, M	First diagnosis	pAML/ M4	4, **15**, 33, 56, 64, **117**	10%	favorable	**DCC:** Kit-M, -I, -K **MLC:** Kit-I **CTX:** Kit-I
**1460**	78, F	First diagnosis	pAML/ M4	**14**, **15**, 34, 56, 117	68%	intermediate	**DCC:** Kit-M, -I, -K **MLC:** Kit-M **CTX:** Kit-M
**1461**	78, M	First diagnosis	BAL	15, 19, 22, 24, 33, **34**, 65	61%	adverse	**DCC:** Kit-M, -I, -K **MLC:** Kit-M, -I, -K **CTX:** Kit-M, -I, -K
**1464**	72, M	First Diagnosis	sAML	13, **34**, 117	50%	–	**DCC:** Kit-M, -I, -K **MLC:** Kit-M, -I, -K **CTX:** Kit-M, -I, -K
**1466**	47, F	First Diagnosis	pAML/ M5	13, 15, 33, **34**, **117**	15%	adverse	**DCC:** Kit-M, -I, -K **MLC:** Kit-M, -I, -K **CTX:** Kit-M, -I, -K
**1468**	66, M	First Diagnosis	pAML	13, 33, 34, **56**, **65**, 117	75%	intermediate	**DCC:** Kit-M, -I, -K
**1489**	55, F	First Diagnosis	pAML/ M0	13, 33, **117**	82%	favorable	**DCC:** Kit-M, -I, -K **MLC:** Kit-M, -I, -K **CTX:** Kit-M, -I, -K
**1621**	71, M	First diagnosis	pAML	**13**, 33, 34, **117**	20%	adverse	**DCC:** Kit-M, -I **MLC:** Kit-M, -I **CTX:** Kit-M, -I
**1622**	49, F	First Diagnosis	pAML	13, **33**, **117**	66%	favorable	**DCC:** Kit-M, -I, -K **MLC:** Kit-M **CTX:** Kit-M
**1623**	67, M	First diagnosis	pAML	4, 7, 24, 33, **34**, **56**	18%	adverse	**DCC:** Kit-M, -I, -K **MLC:** Kit-M **CTX:** Kit-M
**1624**	77, F	First diagnosis	pAML	14, 15, 33, **34**, 64, 56	60%	adverse	**DCC:** Kit-M, -I, -K **MLC:** Kit-M, -I **CTX:** Kit-M, -I
**1625**	60, M	First diagnosis	AML	13, 33, **34**, **117**	11%	intermediate	**DCC:** Kit-M, -I, -K **MLC:** Kit-M, -I **CTX:** Kit-M, -I
**1627**	58, F	First diagnosis	AML	7, 33, **34**, **117**	28%	favorable	**DCC:** Kit-M, -I
**1467**	59, F	Persistent Disease	sAML	13, 33, **34**, 117	31%	–	**DCC:** Kit-M, -I, -K
**1449**	78, M	Relapse	sAML	14, **15**, 33, 34, **56**, 65	62%	–	**DCC:** Kit-M, -I, -K **MLC:** Kit-M **CTX:** Kit-M
**1474**	70, M	Relapse	AML	13, 33, 34, 56, 64, **117**	80%	–	**DCC:** Kit-M, -I, -K
**1463**	60, F	Relapse after HSCT	sAML	2, 3, 13, 14, 19, 33, **34**, 56, 64	30%	–	**DCC:** Kit-M, -I, -K **MLC:** Kit-M **CTX:** Kit-M
**1628**	22, F	Relapse after 2x HSCT	AML	33, **34**, 56, 64, 65, 117	7%	–	**DCC:** Kit-M, -I, -K **MLC:** Kit-M **CTX:** Kit-M

# Patient’s number; h, healthy; F, female; M, male; pAML, primary AML; sAML, secondary AML; BAL, Biphenotypic acute leukemia; FAB, French-American-British classification; M0, Minimally differentiated acute myeloblastic leukemia; M4, acute myelomonocytic leukemia; M5, acute monocytic leukemia; IC, immunocytologically determined; PB, peripheral blood; WB, whole blood; HSCT, hematopoietic stem cell transplantation; DCC, Dendritic cell culture; MLC, mixed lymphocyte culture; CTX, cytotoxicity (fluorolysis) assay; CD, cluster of differentiation; the blasts markers used for expression analysis in each individual patient are highlighted in bold. *ELN Risk Stratification at initial diagnosis. **Various concentrations of response modifiers used.

Mononuclear cells (MNC) were isolated from WB by Ficoll density gradient centrifugation. T-cells were isolated from MNC using the MACS microbead and column based immunomagnetic cell separation technology (Miltenyi Biotec) via positive selection of CD3^+^ cells according to the manufacturer’s instructions (19).

### Immunophenotyping and cell characterization by flow cytometry

Flow cytometric analyses were performed using a FACSCalibur four channel flow cytometer and the CellQuest Pro 6.1 software (Becton Dickinson) to evaluate and quantify frequencies, phenotypes and subsets of leukemic blasts, DCs, monocytes, NK-, CIK and T-cell subtypes, as shown before (9). Abbreviations of all cell types are given in **Table 2**. Flow antibodies for cell staining are outlined in the **Supplementary Data Sheet 2**.

**Table 2:** Cell types evaluated by flow cytometry.

	Name of Subgroups	Surface Marker	Abbreviation Referred to cell subsets	Reference
**Leukemic blast cells**	Blasts	Bla^+^ (CD15^+^, CD14+, CD33+, CD34^+^, CD56^+^, CD65^+^, CD117^+^)	Bla/WB	Schmetzer et al. 2007 (27)
**Dendritic cells**	Proliferating Blasts	Bla^+^DC^-^CD71^+^	Bla_prol_/Bla	Plett 2022 (26)
	Dendritic cells	DC^+^ (CD80^+^, CD83^+^, CD206^+^, CD209^+^)	DC/WB	Schmetzer et al. 2007 (27)
	leukemia derived DC	DC^+^Bla^+^	DC_leu_/WB DC_leu_/DC DC_leu_/Bla	Schmetzer et al. 2007 (27)
	Mature DC	DC^+^CCR7^+^	DC_mat_/WB DC_mat_/DC	Schmetzer et al. 2007 (27)
	Mature DC_leu_	DC^+^Bla^+^CCR7^+^	DC_mat+leu_/WB DC_mat+leu_/DC_leu_ DC_mat+leu_/DC_mat_	Schmetzer et al. 2007 (27)
**Monocytes**	CD14^+^ monocytes	CD14^+^	Mo/WB	Schmetzer et al. 2007 (27)
**T-cells**	CD3^+^pan T-cells	CD3^+^	CD3^+^/WB	Schütti et al. 2024 (19)
	CD4^+^-coexpressing T cells	CD3^+^CD4^+^	CD3^+^CD4^+^/CD3^+^	Schütti et al. 2024 (19)
	CD8^+^-coexpressing T cells	CD3^+^CD8^+^	CD3^+^CD8^+^/CD3^+^	Schütti et al. 2024 (19)
	Naïve T-cells	CD3^+^CD45RO^−^	T_naive_/CD3^+^	Schütti et al. 2024 (19)
	Non-naïve T-cells	CD3^+^CD45RO^+^	T_non-naive_/CD3^+^	Schütti et al. 2024 (19)
	Central (memory) T-cells	CD3^+^CD45RO^+^CCR7^+^	T_CM_/CD3^+^	Schütti et al. 2024 (19)
	Effector (memory) T-cells	CD3^+^CD45RO^+^CCR7^−^	T_EM_/CD3^+^	Schütti et al. 2024 (19)
	CD8^+^-coexpressing non-naive T-cells	CD3^+^CD45RO^+^	CD8^+^T_non-naive_/CD3^+^	Schütti et al. 2024 (19)
	CD8^+^-coexpressing central (memory) T-cells	CD3^+^CD45RO^+^CCR7^+^	CD8^+^T_CM_/CD3^+^	Schütti et al. 2024 (19)
	CD8^+^-coexpressing effector (memory) T-cells	CD3^+^CD45RO^+^CCR7^−^	CD8^+^T_EM_/CD3^+^	Schütti et al. 2024 (19)
	Early proliferating T-cells	CD3^+^CD69^+^	T_prol-early_/CD3^+^	Pepeldjiyska et al. 2022 (43)
	Late proliferating T-cells	CD3^+^CD71^+^	T_prol-late_/CD3^+^	Pepeldjiyska et al. 2022 (43)
	IL-2R^+^IL-7R^low^ expressing T-cells	CD3^+^CD25^+^CD127^low^	T_reg_/CD3^+^	Pepeldjiyska et al. 2022 (43)
	IL-2R^+^IL-7R^low^ expressing CD4^+^T-cells	CD3^+^CD4^+^CD25^+^CD127^low^	CD4^+^T_reg_/CD3^+^ CD4^+^	Pepeldjiyska et al. 2022 (43)
	IL-2R^+^IL-7R^low^ expressing CD8^+^T-cells	CD3^+^CD8^+^CD25^+^CD127^low^	CD8^+^T_reg_/CD3^+^ CD8^+^	Pepeldjiyska et al. 2022 (43)
**B-cells**	B-cells	CD19^+^	B/WB	Schütti et al. 2024 (19)
**CIK-cells**	Cytokine-induced killer cells	CD3^+^CD56^+^	CIK/WB	Schütti et al. 2024 (19)
**NK-cells**	Natural killer cells	CD3^−^CD56^+^	NK/WB	Schütti et al. 2024 (19)

### Dendritic cell culture

**Figure 1:**
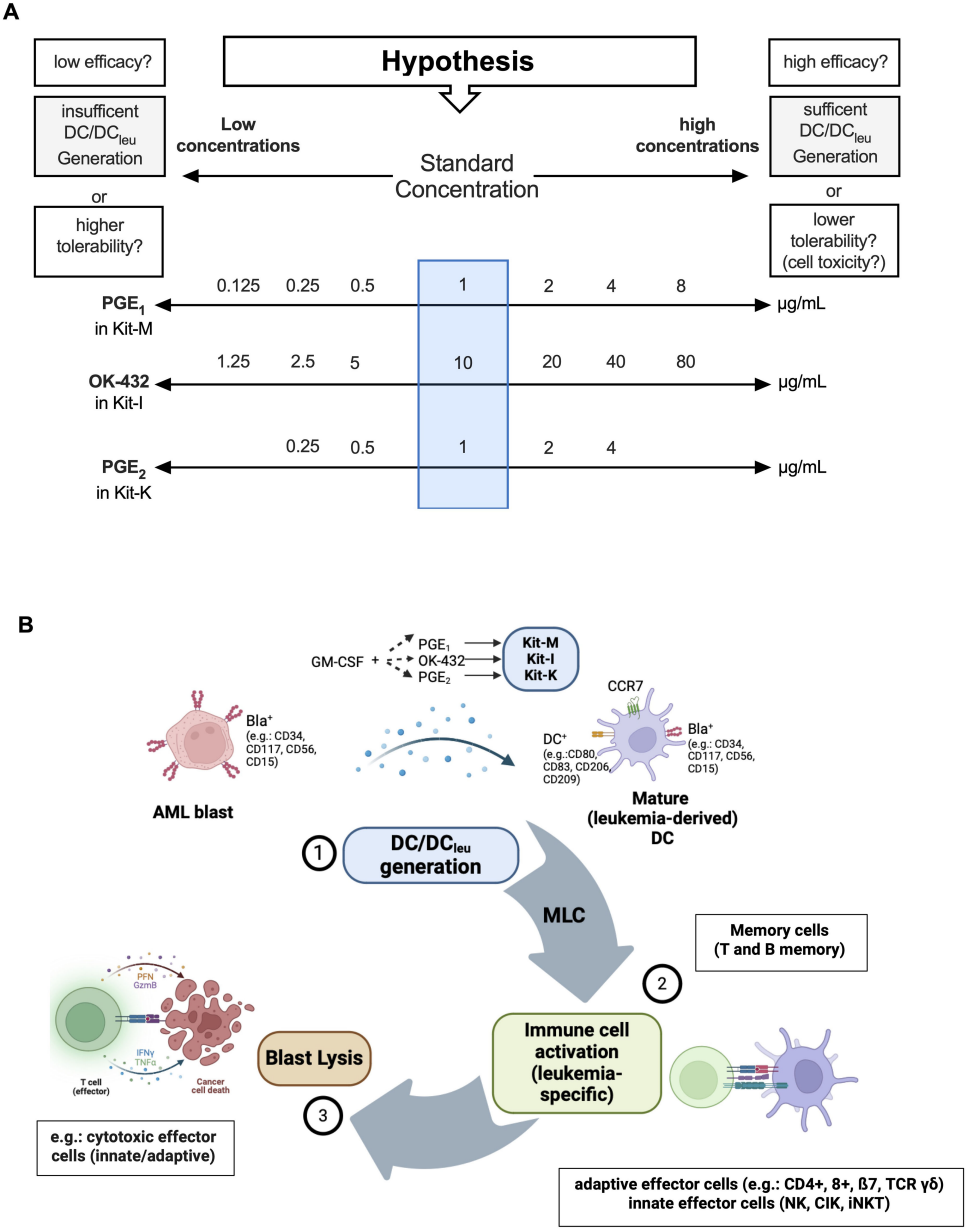
DC/DC_leu_ generation using different (concentrations of) response modifiers and mode of action of DC/DC_leu_-mediated antileukemic reactions. **(A)** Overview of the varying concentrations of response modifiers (PGE_1_, OK-432, PGE_2_) in addition to GM-CSF (800 U/ml) within DC/DC_leu_-generating kits. These were used to define the optimal concentration for each Kit to generate sufficient DC fractions in WB without off-target cell toxicity. **(B)** A schematic illustration of Kit induced DC/DC_leu_ mediated blast lysis: DC/DC_leu_ were generated from blast containing AML whole blood (WB), followed by T cell enriched mixed lymphocyte culture (MLC) and a functional cytotoxicity assay.

Immunomodulators were added to WB cultures as previously described (18). A culture without response modifiers served as negative control. Cells were harvested after 7-9 days. For AML DC cultures and DC cultures from healthy WB, we tested varying concentrations of PGE_1_ (0.125-8.0 μg/ml), OK-432 (1.25-80 μg/ml), and PGE_2_ (0.25-4.0 μg/ml). To study GM-CSF-independent differences of DC/DC_leu_ generation and anti-leukemic functionality, a constant concentration of GM-CSF (800 U/ml) was applied across all protocols. The composition of DC/DC_leu_ generating protocols (Kit-M, -I, -K) including the specific concentrations of the response modifiers are provided in **Table 3** and **Figure 1A**.

**Table 3:** DC/DC_leu_-generating protocols with Kits.

DC/DC_leu_-Generating Protocols	Composition	Standard Concentration	Time of stimulation/ restimulation	Mode of action	Culture time	References
**KIT-M**	GM-CSF PGE_1_	800 U/ml 1 μg/ml	day 0/ day 2-4	**GM-CSF:** induction of myeloid (DC-) differentiation **PGE_1_:** danger signaling, stimulation of DC-maturation and migration (via CCR7 expression) **OK-432:** a penicillin-killed lyophilized streptococcal agent, danger signaling via TLR4, stimulation of DC-differentiation **PGE_2_:** s danger signaling, stimulation of DC-maturation and migration (via CCR7 expression)	7-9 days	Schwepcke et al 2022 (18) European Patent No 15801987.7–1118 US Patent NO 10912820 (Modiblast GmbH)
**KIT-I**	GM-CSF OK-432	800 U/ml 10 μg/ml (0,1 KE)	day 0/ day 2-4		7-9 days	
**KIT-K**	GM-CSF PGE_2_	800 U/ml 1 μg/ml	day 0/ day 2-4		7-9 days	

GM-CSF, granulocyte macrophage colony stimulating factor; PGE_1_, Prostaglandin E_1_; PGE_2_, Prostaglandin E_2_; OK-432, Picibanil; TLR4, toll- like receptor 4; U, Unit; KE, Klinische Einheit (a unit for OK-432 doses); CCR7, chemokine (C-C motif) receptor.

Flow cytometric analyses of leukemic blasts, DC, DC_leu_ and DC_mat_ followed a refined gating strategy (9, 26, 27). DC_leu_ were analyzed by the co-expression of at least one blast marker including lineage-aberrant markers (e.g., CD117) and at least one DC marker not expressed on naïve blasts (e.g., CD80). Mature DC/DC_leu_ were assessed by examining the co-expression of CCR7 on DC or DC_leu_. A schematic overview of the experimental strategy for DC/DC_leu_ generation and flow cytometric analysis plan for identifying DC_leu_ is demonstrated in **Figure 1B**.

### Mixed lymphocyte culture

DC/DC_leu_ containing Kit treated WB culture (DCC) from dendritic cell culture were used to stimulate immune cells in T-cell enriched MLC as shown before (10, 19).

### Cytotoxicity fluorolysis assay

Blast lytic activity of T-cell enriched immunoreactive cells was measured after MLC with Kit treated WB-cultures. To this end, a fixed fraction of MLC containing 1×10^6^ T-cells (as effector cells) and 1×10^6^ thawed autologous leukemic blasts (as target cells) was employed. As a control, effector and target cells were cultured under the same conditions but separately and only combined prior to flow cytometric analyses. The achieved blast lytic activity was defined as the percentual difference of viable 7AAD negative target cells (blasts) between the cocultured vs not cocultured effector/target cells (9). Cytotoxic effects against T-cells, labeled as target cells, were analyzed in order to quantify potential T-cell toxic effects.

### Statistical methods

Data are presented as mean ± 95% confidence intervals, standard deviation (SD) or standard error of mean (SEM). Statistical tests are provided in figure legends (Wilcoxon matched paired signed rank test and Wilcoxon rank-sum test for standard concentrations, Bonferroni’s multiple comparisons test and Tukey’s multiple comparison test for concentrations, Spearman’s test for correlation analyses). Statistical significance was defined as ‘not significant’ (p>0.10), ‘borderline significant’ (*p<0.1), ‘significant’ (**p<0.05), ‘very significant’ (***p<0.01), or ‘highly significant’ (****p<0.001). Statistical analyses and figures were implemented using Prism 10.4.0 (GraphPad Software) and “bioRender.com”.

## Results

### Increased generation of (mature) DCs from healthy WB with standard concentrations of Kit-M, Kit-I and Kit-K, but not with single response modifiers alone

**Figure 2:**
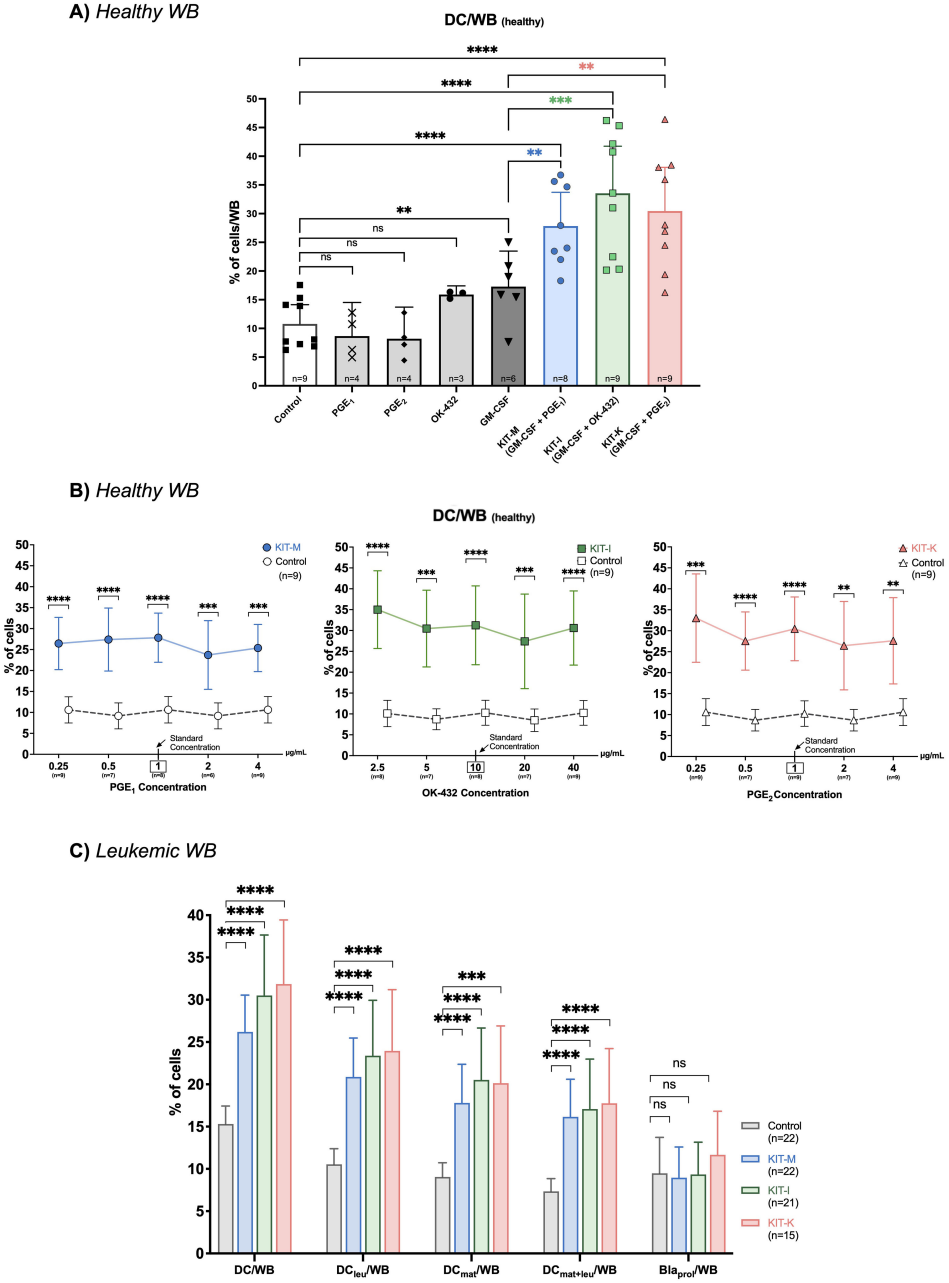
DC-generation using single or combined response modifiers. Frequencies of generated DCs from healthy **(A, B)** or leukemic WB **(C)** following treatment with different response modifying agents. **(A)** DC generation using either single response modifiers or Kits in standard concentrations of GM-CSF and PGE_1_ (Kit-M: 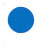), GM-CSF and OK-432 (Kit-I: 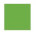), or GM-CSF and PGE_2_ (Kit-K: 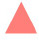). **(B)** DC generation using Kits with fixed standard concentrations of GM-CSF (800 U/mL) and varying concentrations of PGE_1_ (Kit-M), OK-432 (Kit-I), and PGE_2_ (Kit-K) (from left to right). A box and arrow indicate the respective standard concentration. **(C)** Generation of DC subsets from leukemic WB with standard concentrations of Kit-M, Kit-I and Kit-K vs. control. Abbreviations of DC cell subtypes are given in **Table 2**. Data are presented as mean and 95% confidence intervals. Wilcoxon rank-sum test, Bonferroni’s and Tukey’s multiple comparison test were performed to calculate statistics, ****p<0.001, ***p<0.01, **p<0.05, *p<0.1 borderline significant, p>0.1 not significant (ns).

Compared to a control without response modifiers, we could generate significantly higher frequencies of (mature) DCs from healthy WB with immunomodulatory Kits in standard concentrations including Kit-M (GM-CSF, PGE_1_), Kit-I (GM-CSF, OK-432), and Kit-K (GM-CSF, PGE_2_) (**Figure 2A**). However, we did not observe increased (mature) DC generation compared to control when cells were stimulated with single response modifiers.

To address whether DC-generating effects are predominantly driven by GM-CSF alone, we compared the frequencies of generated DCs induced by only GM-CSF with the Kit-M, Kit-I and Kit-K groups. Indeed, we noted significantly higher frequencies of (mature) DCs were generated from healthy WB with the immunomodulatory Kits in standard concentrations compared to GM-CSF alone (**Figure 2A**).

### Increased generation of (mature) DCs from healthy WB with GM-CSF combined with various combinations and concentrations of PGE_1,_ PGE_2_ or OK-432

Next, we evaluated how the concentrations of immunomodulatory agents within each Kit impact DC generation. We added five different concentrations of PGE_1_, PGE_2_ or OK-432 to DC-cultures from healthy WB, while maintaining a constant GM-CSF concentration. Compared to control, significantly more DCs could be generated from healthy WB with PGE_1_ and PGE_2_ at concentrations ranging between 0.25-4 μg/mL (for Kit-M and Kit-K), and for OK-432 concentrations ranging between 2.5-40 μg/mL (for Kit-I). While we noted notable differences in the frequencies of generated DCs compared with control, we did not detect a significant difference when comparing the different response modifier concentrations against each other (*p >*0.1 for each cross-concentration comparison). The respective percentual differences in DC-frequencies vs. control are outlined in **Figure 2B**. Similarly, we could generate significantly higher frequencies of mature DCs (DC_mat_) and monocyte derived DCs (Mo-DC) at the same concentration ranges compared to control (**Supplementary Figure S1**).

### Increased generation of mature and leukemia-derived DCs from leukemic WB across multiple immunomodulatory Kits

We were able to generate significantly higher frequencies of DCs and specific DC subtypes (e.g. DC_leu_, DC_mat_, DC_mat+leu_) from leukemic WB with all three immunomodulatory kits compared to the control without concurrent induction of blast proliferation (**Figure 2C**, **Supplementary Figure S2**) – using previously established standard concentrations (26). For example, we noted an approximately two-fold increase in the generation of (mature) DC_leu_s compared to the control for each of the kits (**Figure 2C**, left). Furthermore, the relative frequencies of DC, DC_leu_, and DC_mat+leu_ and their subsets did not significantly differ between Kits.

### Increased generation of DC-subsets from leukemic WB according to concentrations of PGE_1,2_ or OK-432

**Figure 3:**
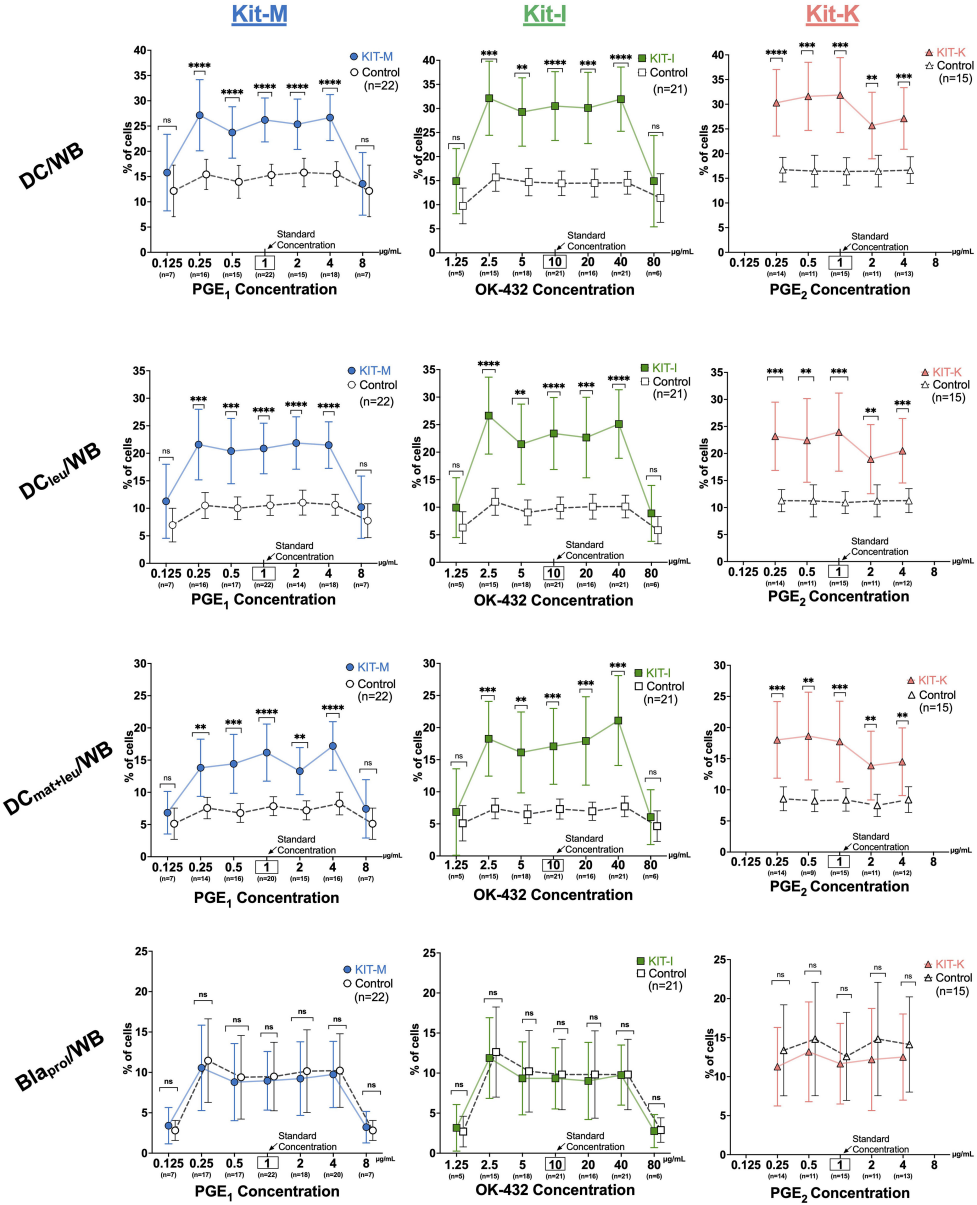
Generation of DC subsets from leukemic whole blood with varying response modifier concentrations. From left to right: Frequencies of DC subsets and proliferating blasts in leukemic whole blood (WB) for Kit-M (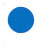), Kit-I (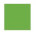), Kit-K (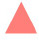) using a constant concentration of GM-CSF (800 U/mL) and varying concentrations of PGE_1_, OK-432 and PGE_2_, respectively. Abbreviations of cell subtypes are provided in **Table 2**. Data are presented as mean ± 95% confidence intervals. Bonferroni’s and Tukey’s multiple comparisons tests were performed to calculate statistics, p-values are shown above the line graphs, ****p <0.001, ***p<0.01, **p<0.05, *p<0.1 borderline significant, p>0.1 not significant (ns).

To assess how changes of response modifier concentrations impact DC generation from leukemic WB, we analyzed seven different concentrations of PGE_1_ (0.125-8 μg/mL) and OK-432 (1.25-80 μg/mL) and five different concentrations of PGE_2_ (0.25-4 μg/mL), while maintaining a constant concentration of GM-CSF (800 U/mL). For PGE_1_ (Kit-M), the relative frequencies of (mature) DCs, DC_leu_, and DC_mat+leu_ were significantly increased compared to control across five different concentrations (0.25 to 4 μg/mL) (**Figure 3**, left). Conversely, very low (0.125 μg/mL) or very high (8 μg/mL) concentrations of PGE_1_ did not give rise to increased DC values. Similar findings were noted for OK-432 (Kit-I) with optimal generation of DCs and DC-subsets at intermediary concentrations of 2.5-40 μg/mL OK-432, whereas very low or high (1.25 or 80 μg/mL) OK-432 concentrations did not yield increased DC values (**Figure 3**, middle). Of interest, we found that the DC_leu_ (but not DC_mat_) numbers within the generated DCs were significantly increased relative to control for the higher (80 μg/mL, *p* = 0.04) but not lower OK-432 concentrations (1.25 μg/mL*, p* > 0.9), as outlined in **Supplementary Figure S3**. Additionally, the higher relative ratio of DC_leu_ to blasts was maintained with the higher concentrations of OK-432 (80 μg/mL) in Kit-I. For PGE_2_ (Kit-K), lower concentrations between 0.25-1 μg/mL yielded the highest frequencies of DCs and DC subsets (**Figure 3**, right). Nonetheless, higher concentrations of PGE_2_ still showed increased DC values compared to the control. Importantly, none of the kits resulted in increased blast frequencies relative to control, irrespective of the applied concentrations of the response modifiers (**Figure 3**, lowest row). Comparable findings for all three Kits and varying concentrations of the response modifiers were found for further DC-subtypes (DC_leu_/DC, DC_mat+leu_/DC, DC_leu_/Bla, **Supplementary Figure S3**).

### Kit pretreated stimulator cell fractions containing mature DC/DC_leu_ upregulate activated and memory T-cells while downregulating regulatory T-cells

**Figure 4:**
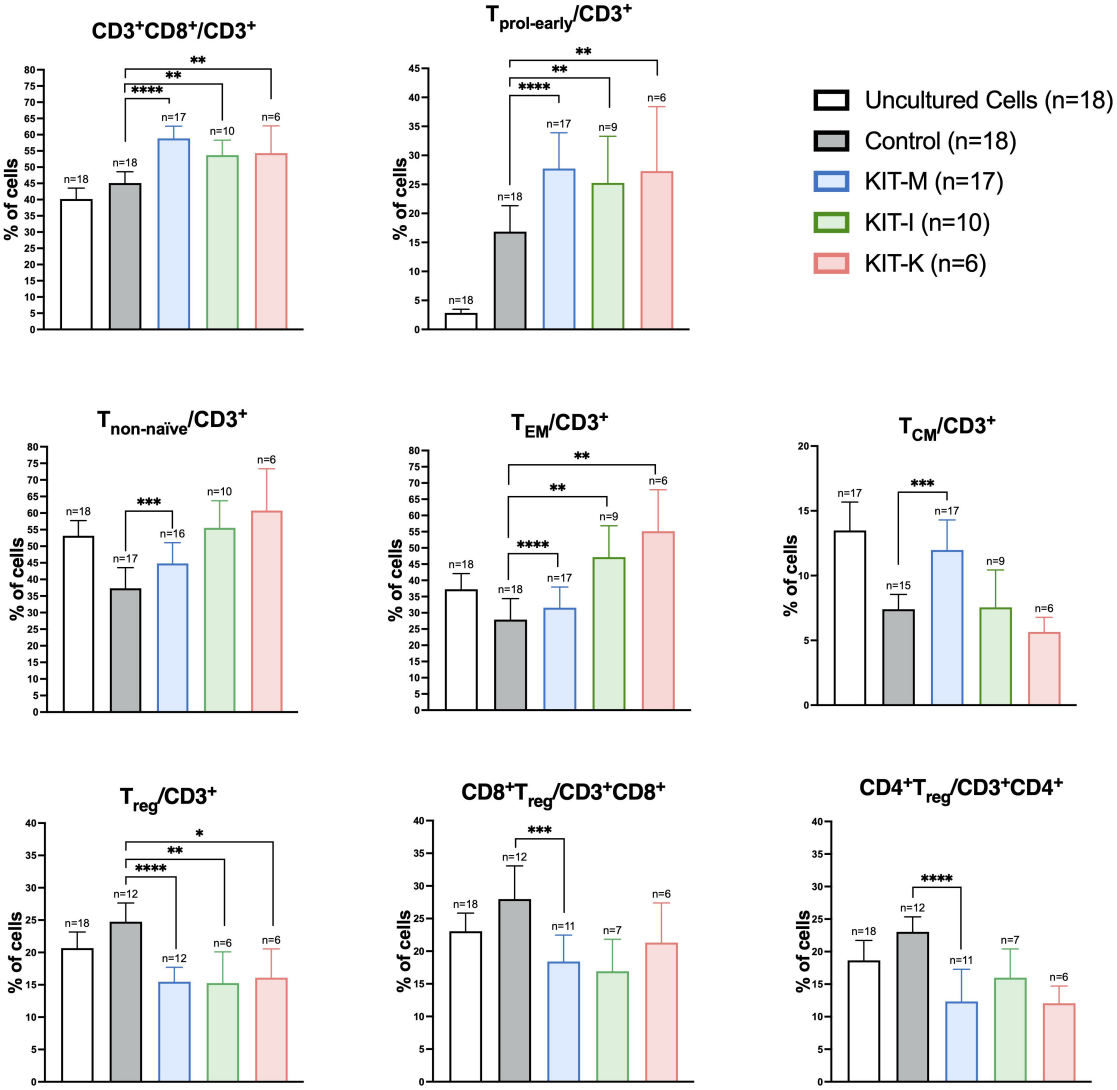
Kit-treated leukemic whole blood in mixed lymphocyte culture. Frequencies of T-cell subsets before (uncultured cells) and after T-cell enriched mixed lymphocyte culture (MLC) with Kit-treated or untreated (control) whole blood. Standard concentrations of PGE_1_, OK-432 and PGE_2_ were applied for Kit-M, -I, respectively (in addition to GM-CSF). Data are presented as mean ±SEM. Wilcoxon matched paired signed rank test was performed to calculate statistics, ****p <0.001, ***p<0.01, **p<0.05, *p<0.1 borderline significant, p>0.1 not significant (ns). Abbreviations of cell subtypes are given in **Table 2**.

To further assess the DC/DC_leu_ stimulating effects on immunoreactive cells in the presence of IL-2, we compared T-cell subtype compositions in CD3+ T-cell fractions before (uncultured cells) and after T-cell enriched MLC with Kit treated vs. untreated WB (MLC_Control,_ MLC_KIT-M_, MLC_KIT-I_, MLC_KIT-K_). As previously demonstrated (9, 19), we found significantly increased frequencies of activated (proliferating, non-naïve) and memory (T_EM_, T_CM_) T-cells, but reduced frequencies of regulatory T-cells (T_reg_) in Kit pretreated vs. non-pretreated settings (**Figure 4**). Of interest, we only observed an increase of T_CM_ cells with Kit-M (~two-fold increase). In addition, downregulation of CD4+ and CD8+ T_reg_ cells was restricted to Kit-M, although this observation may have been facilitated by lower case numbers for Kit-I and Kit-K.

### Increased frequencies of activated and memory T cell subsets after MLC with Kit pretreated leukemic WB are dependent on concentrations of PGE_1,2_ or OK-432

**Figure 5:**
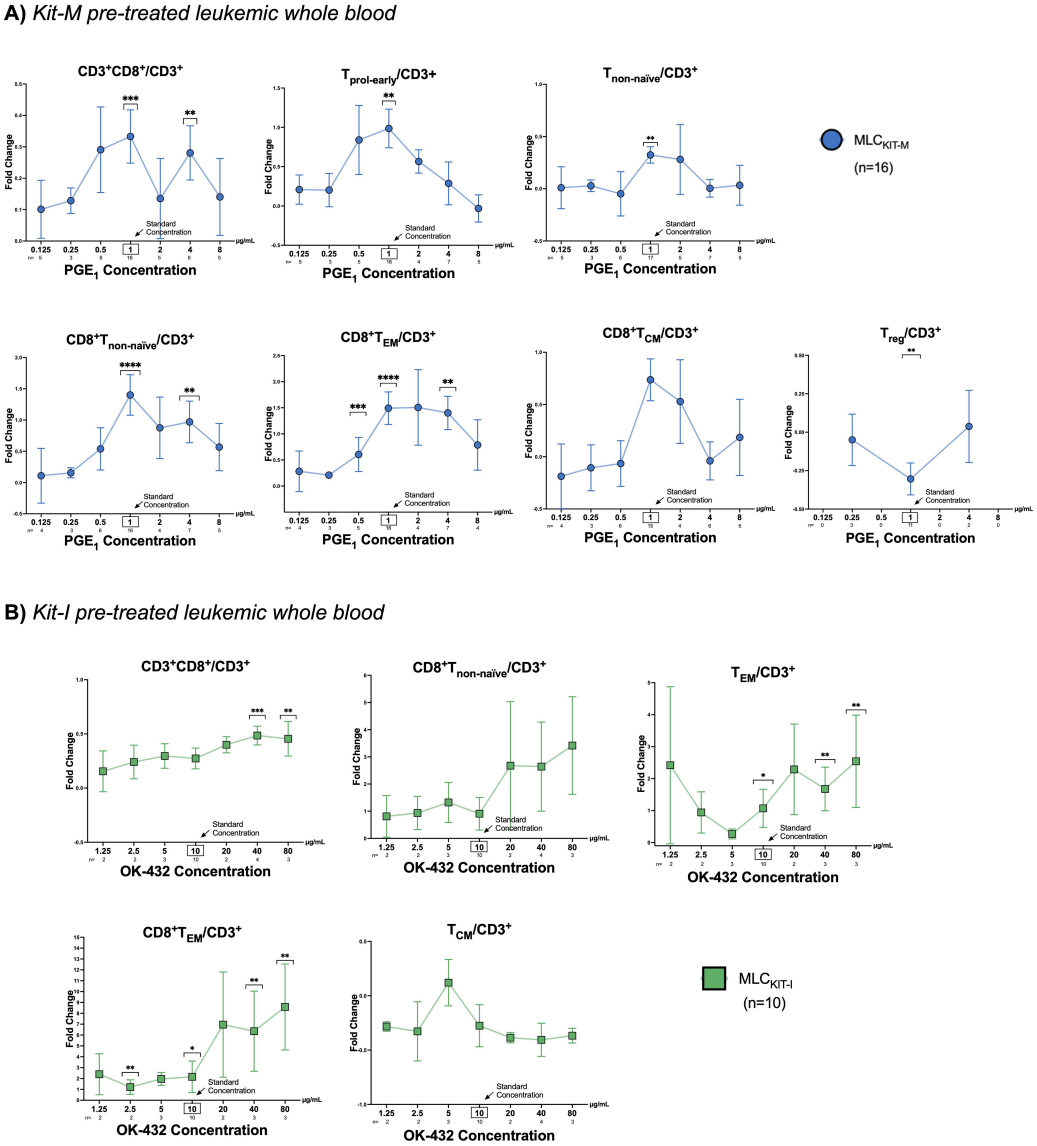
T-cell composition following mixed lymphocyte culture of Kit pre-treated and untreated leukemic whole blood. **(A, B)** Fold changes of frequencies of T cell subtypes in Kit pre-treated compared to non-Kit-pretreated leukemic WB samples using constant concentrations of GM-CSF and **(A)** varying concentrations of PGE_1_ (0.125, 0.25, 0.5, 1, 2, 4, 8 µg/ml) or **(B)** OK-432 (1.25, 2.5, 5, 10, 20, 40, 80 µg/ml). Data are presented as mean ±SEM. Bonferroni’s multiple comparisons test were performed to calculate statistics, ****p <0.001, ***p<0.01, **p<0.05, *p<0.1 borderline significant, p>0.1 not significant (ns). Abbreviations for cell subtypes are given in **Table 2**.

Next, we analyzed T-cell subtypes in Kit pretreated vs untreated leukemic WB (used as stimulator cells in MLC) in the context of varying concentrations of PGE_1_, PGE_2_ or OK-432 (**Figure 1**). Representative flow cytometry scatter plots and the gating strategy to identify activated and memory T-cell subsets are outlined in **Supplementary Figure S4**. Compared to control samples that were not pretreated with Kit-M, we noted a clear or even significant increase of the frequencies of proliferating, non-naïve, and memory T-cells (especially of CD8+ subtypes) for PGE_1_ concentrations ranging between 0.5-4 μg/mL. The respective fold changes in the frequencies of the different T-cell subtypes with Kit-M compared to untreated controls are outlined in **Figure 5A**. For Kit-M, the most notable fold changes were observed around the previously established standard concentration (1 µg/mL). In addition, we noted an inversion of the CD4+ to CD8+ ratio (in favor of CD8+) with Kit-M compared to control, and a particular increase of (CD8+) T_EM_ relative to T_CM_ at higher PGE_1_ concentrations (**Supplementary Figure S5**).

When examining T-cell subsets following MLC of Kit-I pre-treated leukemic WB, we found a clear or even significant increase of proliferating, activated or memory T-cells (especially of CD8+ subtypes) in direct correlation with higher OK-432 concentrations. Fold changes of the frequencies of particular T-cell subtypes compared to controls without pretreatment of response modifiers are given in **Figure 5B**. For Kit-I, the highest frequencies of activated T-cells were noted in concentration ranges above the standard concentration for OK-432 (i.e., above 10 µg/mL). Data with varying concentrations of PGE_2_ (Kit-K) could not be generated due to low sample numbers.

### Improved blast lytic activity in a cytotoxicity assay with DC/DC_leu_ stimulated T- and immune cells

**Figure 6:**
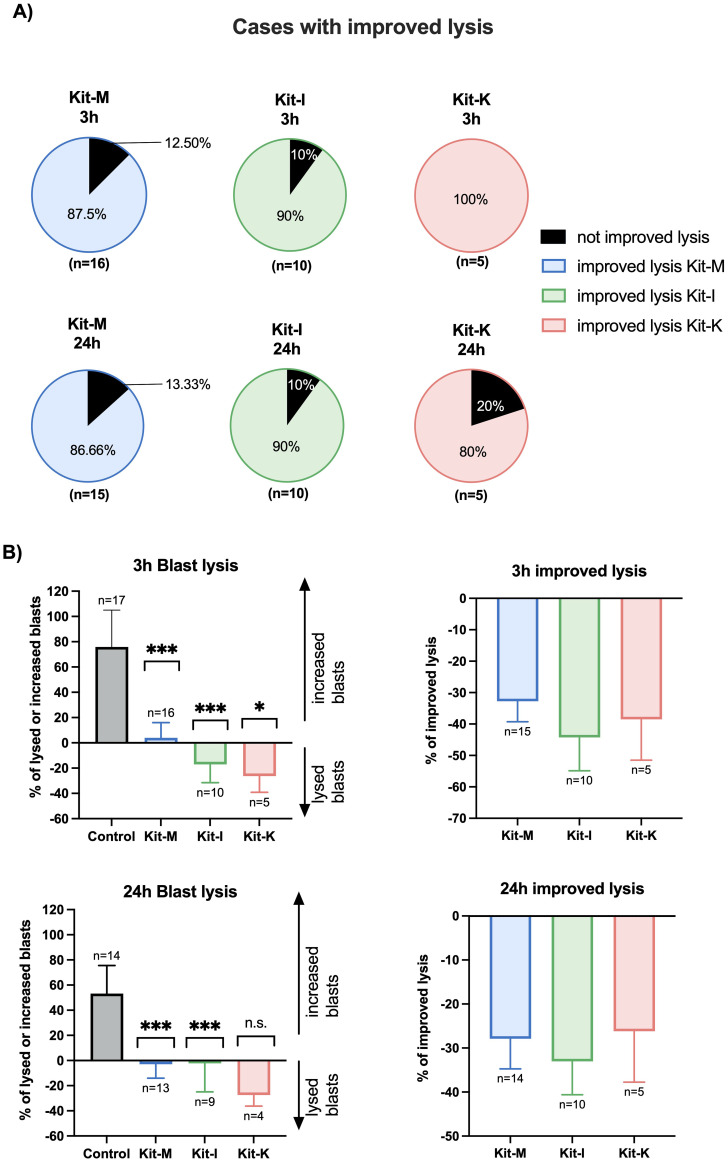
Stimulatory effects of Kit-treated leukemic whole blood on the anti-leukemic activity of immunoreactive cells following MLC in a cytotoxicity assay. **(A)** Percentage of cases with improved blast lysis with Kit-M, -I and -K pretreated cells and untreated control following 3h and 24h of coculture of these effector cells with blast target cells. Standard concentrations of response modifiers were used for each Kit. **(B)** Average of lysed/increased blasts (left side) and improved blast lysis compared to control (right side). Data are presented as mean ±SEM. Wilcoxon matched paired signed rank test was performed to calculate statistics, ****p <0.001, ***p<0.01, **p<0.05, *p<0.1 borderline significant, p>0.1 not significant (ns).

To assess blast lytic activity secondary to DC/DC_leu_ stimulated T- and immune cells after MLC, we next performed cytotoxicity fluorolysis assays. Overall, we found improved blast lysis compared to the control for more than 80% of cases when using standard concentrations of Kit-M, -I, -K (**Figure 6A**) – consistent with prior findings (18, 19). The improved blast lysis after 3h and 24h of Kit-M, -I, -K pretreated samples vs control following MLC against blast target cells is provided in **Figure 6A**. The frequencies of lysed/increased blasts as well as improved blast lysis in all Kit treated samples compared to control are outlined in **Figure 6B**, confirming previous data (18).

At 3 hours, there was a statistically significant increase in blast lysis compared to the control across all three kits (**Figure 6B**, top left). Relative to the respective control sample, the highest improvement in blast lysis at 3 hours was observed for Kit-I (median 42% improvement in blast lysis), followed by Kit-K (38% improvement) and Kit-M (32% improvement) (**Figure 6B**, top right). At 24 hours, we confirmed reduced blast proliferation for the Kit-treated samples (**Figure 6B**, bottom left). Furthermore, all three Kits displayed improved blast lytic activity at 24 hours compared to control, which was highest for Kit-I (**Figure 6B**, bottom right).

### Antileukemic cytotoxicity after MLC of Kit-treated (vs untreated) leukemic WB depends on response modifier concentrations

**Figure 7:**
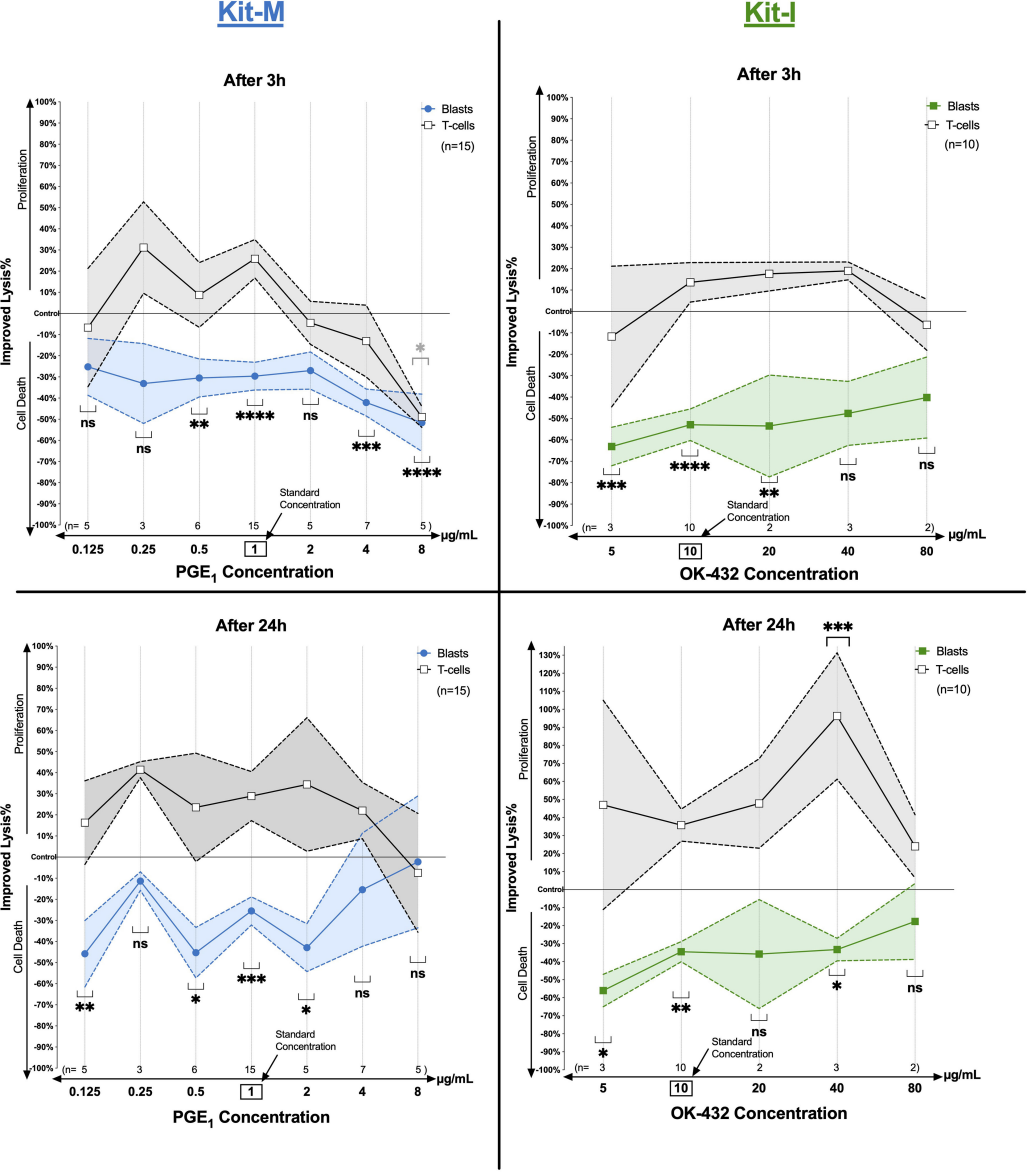
Anti-leukemic activity in a cytotoxicity assay according to varying response modifier concentrations. Stimulatory effects of Kit treated vs untreated leukemic whole blood (WB) using different concentrations of PGE_1_ or OK-432 and constant dose of GM-CSF in Kit-M or Kit-I on the anti-leukemic and anti-T cell activity of immunoreactive cells after MLC, as measured in a cytotoxicity assay (CTX). Provided are the percentages of improved blast lysis/proliferation and T-cell lysis/proliferation with Kit-M or Kit-I pre-treated vs untreated cells (after MLC) after 3h and 24h of co-culture of these ‘effector cells’ with blast target cells, respectively. Data are represented as mean ±SEM. Bonferroni’s multiple comparisons test were performed to calculate statistics, ****p <0.001, ***p<0.01, **p<0.05, <*p<0.1 borderline significant, p>0.1 not significant (ns).

To examine how different concentrations of response modifiers influence the antileukemic cytotoxicity propagated by the immunomodulatory kits, we performed cytotoxicity fluorolysis assays quantifying the improvement of blast lysis (vs control) in samples pretreated with varying concentrations of PGE_1_ (Kit-M: **Figure 7**, left) or OK-432 (Kit-I: **Figure 7**, right). For Kit-M, we identified significantly increased blast lysis compared to controls for PGE_1_ concentrations of 0.5-2 μg/mL following coincubation of effector with target cells for 3 hours and 24 hours, respectively (**Figure 7**, left). While we also noted increased blast lysis after 3 hours at higher PGE_1_ concentrations (4-8 µg/mL), this was accompanied by coincident death of T-cells. Moreover, diminished blast lysis and decreased T-cell proliferation at higher PGE_1_ concentration ranges was confirmed for Kit-M after 24 hours. In contrast, a direct positive correlation of increasing OK-432 concentrations (between 5-40 μg/mL) with improved blast lysis was seen without decreasing T-cell proliferation for Kit-I pretreated samples (**Figure 7**, right). Of interest, we noted a particular increase in T-cell proliferation at a higher OK-432 concentration of 40 µg/mL, which corresponded to high generation of DCs and DC subtypes with Kit-I (**Figure 3**) and was accompanied by significant blast lysis after 24 hours.

### The frequencies of mature DC_leu_ and activated T-cell subtypes associate with improved blast lysis in a concentration-dependent manner for PGE_1_ and OK-432

**Figure 8:**
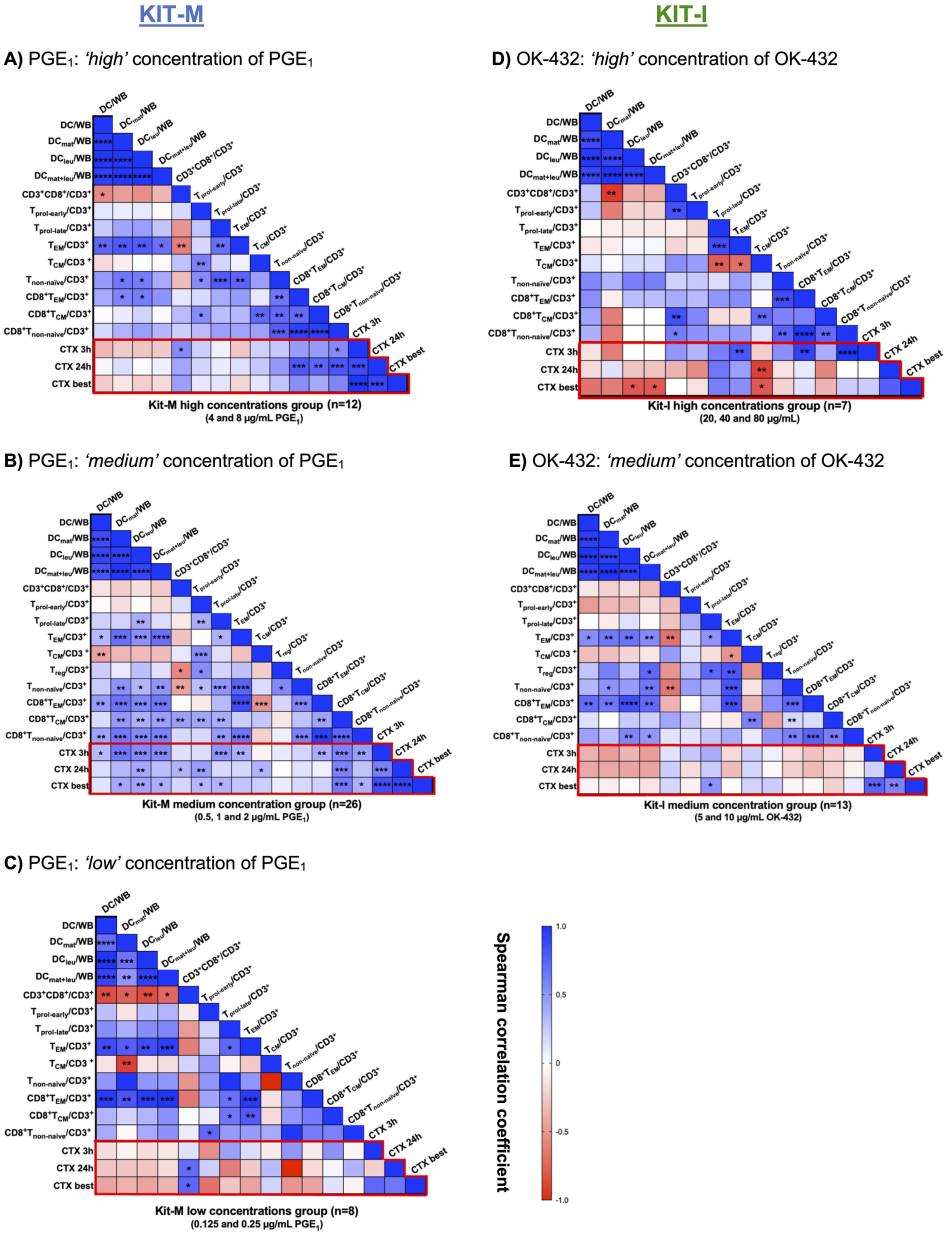
Correlation analyses of generated DC and T-cell subsets with improved lysis in the cytotoxicity assay. **(A–C)** Leukemic whole blood (WB) treated with high **(A)**, medium **(B)** or low **(C)** concentrations of PGE_1_ in Kit-M. **(D, E)** Leukemic WB treated with high **(D)** or medium **(E)** concentrations of OK-432 in Kit-I. The heat map analyses demonstrate the correlation between DC and T-cell subtypes (generated with Kit-M and Kit-I) and improved blast lysis (CTX assay) after 3 and 24 hours or choosing the best achieved improved lysis (CTX best). Spearman correlation tests were performed. Heatmap colors indicate positive (blue) vs. negative (red) correlation coefficients. Respective p values are shown for each comparison in the individual boxes ****p <0.001, ***p<0.01, **p<0.05, 0.05<*p<0.1 borderline significant, p>0.1 not significant (ns).

To understand the association between Kit-mediated generation of DCs and activation of T cell subsets with the observed anti-leukemic effects, we performed a correlation analysis using the results from the cytotoxicity assay as the primary endpoint (**Figure 8**, red box). Based on the outcomes of the cytotoxicity assay, we aggregated results according to high, medium and low concentration ranges for PGE_1_ (**Figures 8A–C**) and OK-432 (**Figures 8D, E**).

Notably, we demonstrated (highly) significant correlations between the frequencies of (mature) DC_leu_ and (CD8+) non-naïve T-cells and T_EM_/T_CM_ in the ‘medium’ PGE_1_ concentration group (0,5-2 μg/mL), which were less pronounced in the ‘high’ (4-8 μg/mL) or the ‘low’ (0.125-0.25 μg/mL) PGE_1_ concentration groups (**Figures 8A–C**). Compared to the low and high concentration groups, we noted more extensive positive correlations between (mature) DC_leu_ with activated T-cell populations in the ‘medium’ PGE_1_ concentration group. Moreover, the increased frequencies of (mature) DC_leu_ correlated with improved blast lysis in the ‘medium’ PGE_1_ group (**Figure 8B**).

We demonstrated (highly) significant correlations between the frequencies of (mature) DC_leu_ and (CD8+) non naïve T cells and T_EM_ in the ‘medium’ (5-10 μg/mL), but not the ‘high’ OK-432 concentration group (20-80 μg/mL) (**Figures 8D, E**). We did not find a significant association between (mature) DC_leu_ generation and improved blast lysis for both concentration groups. However, a positive association was noted for (CD8+) T_EM_ and T_non-naïve_ with improved blast lysis in the ‘high’ OK-432 concentration group (**Figure 8D**), while a negative association was observed between T_CM_ and improved blast lysis.

## Discussion

In this preclinical study, we observed that DC/DC_leu_ can be generated with three different immunomodulatory kits (e.g., Kit-M, -I, -K) from both healthy and AML whole blood and identified optimal *ex vivo* drug concentrations for efficient DC generation. After stimulation of immune cells in mixed lymphocyte culture with DC/DC_leu_ containing Kit-treated whole blood, we observed specific patterns of immune cell and T-cell activation. Importantly, this translated into improved anti-leukemic activity and abrogation of blast proliferation.

### Current therapeutic landscape of DC-based immunotherapy

Dendritic cells (DCs) serve as one of the most influential facilitators within the immune system, acting as a bridge between the innate and adaptive immune system (28). These professional antigen presenting cells (APCs) possess the capacity to migrate into different tissues, can induce an immunological memory, and act as key initiators of tumor-specific immune responses. Because of these attributes, multiple strategies have been developed to target and/or utilize DCs for cancer immunotherapy, including the administration of antigens with immunomodulators that mobilize and activate endogenous DCs, as well as the generation of DC-based vaccines (28). Of interest, DCs can also play an important role in mediating host responses to other promising immunotherapies, as was demonstrated for chimeric antigen receptor (CAR) T-cells in refractory solid tumors (29). Thus, DCs can be readily combined with other treatment modalities to enhance tumor-reactive lymphocyte populations (30, 31).

### Challenges for immunotherapies in AML

Given the lack of immunogenicity of AML blasts due to the inherently low tumor mutational burden (32) and the on-target/off-tumor expression of leukemia-associated antigens (LAAs) on non-leukemic myeloid cells, novel antigen-directed immunotherapies like bispecific antibodies or CAR T-cell therapy face significant hurdles in effectively targeting leukemic cells (33). Furthermore, these therapies are associated with a unique toxicity profile including cytokine release syndrome (CRS), neurotoxicity (ICANS), hematotoxicity and infectious complications (34–37). Due to the complex manufacturing procedures, they also exhibit relevant logistic and technical challenges and carry a high financial strain, limiting their broad use. Alternative treatment options are thus needed which are i) not restricted to specific LAAs and ii) easy-to-apply technically and logistically.

### DC-based treatment for AML

Over recent decades, various methodologies have been devised to leverage DCs as a therapeutic approach for AML – a notoriously difficult disease to treat. Treatment of AML patients with manipulated DCs (loaded with leukemic antigens) has already shown promising effects with respect to inducing leukemia-specific reactions *in vivo*, resulting in subsequent stabilization of disease remissions (5, 30, 38, 39). However, the disadvantages of these approaches lie in the work- and cost-intensive production of manipulated DCs under GMP conditions, followed by the logistically challenging adoptive transfer of cells to patients (40). In contrast, our approach intends to convert (residual) blasts within the patients’ body to DC_leu_, thereby activating the immune system against the patients’ entire leukemic antigen repertoire directly *in vivo*. To this end, we have developed ‘Kits’ that contain (clinically approved) response modifiers, which generate DC/DC_leu_ from leukemic WB and, moreover, hold the distinct ability of inducing antileukemic reactions following stimulation of immune cells in mixed lymphocyte culture (9, 19). In previous work, we could select the three Kits that best mediate antileukemic reactions (Kit-M/-I/-K) (18). In addition, we could demonstrate that Kit-I and -M exhibit superior capacity to induce antileukemic reactions (i.e., blast reduction) in leukemia-diseased rats (7). Notably, three therapy-refractory patients treated with Kit-M in an off-label rescue treatment were shown to produce leukemia-specific immune cells, accompanied by a decrease, or at least a stabilization, of the peripheral blast count [Anand, personal communication and (7)].

### Concentration-dependent DC/DC_leu_ generation and immune cell activation

Our data show, that PGE_1_ leads to increased (DC/DC_leu_ mediated) anti-leukemic *ex vivo* reactions in ‘medium’ but not in ‘low’ or ‘high’ concentrations (**Figures 4**, **8**). High concentrations of PGE_1_ might even lead to T-cell-toxic effects (**Figure 8**). This would be consistent with the sensitivity of dendritic cells to immunometabolic and cytokine-mediated stressors, which can result in profound metabolic reprogramming (41). On the other hand, OK-432 added in various concentrations to leukemic WB samples (in addition to GM-CSF) did not exhibit as prominent off-target T-cell toxic effects, which can be interpreted in the context of the different modes of action of Kit-I vs Kit-M (e.g., favoring innate immunity) – as discussed in previous studies (9, 18). Importantly, in-depth correlation analyses supported our findings: only the ‘medium’ concentrations of PGE_1_ in Kit-M showed a strong correlation between DC subtypes and activated T cells, including reduced regulatory and induced memory T cells, which was also accompanied by significantly improved blast lysis. Accordingly, an optimal or homeostatic balance of T_EM_ to T_CM_ (and CD4 to CD8 T cells) may be critical to provide an effective pro-inflammatory immune milieu without resulting in excessive cytokine-mediated cytotoxicity. It should be noted that high concentrations of OK-432 (as high as 40 µg/mL) did not result in as extensive off-target cytotoxicity (**Figure 8**), highlighting differences in the concentration-dependent nature of immune cell activation compared to PGE_1_. With respect to Kit-K, our data might point to suboptimal generation of DC (subtypes) above 2 µg/mL, although data regarding the functional significance are missing due to low cell counts.

### Anti-leukemic activity and clinical outlook

With respect to the further clinical development of Kit-based DC/DC_leu_-inducing treatment strategies, our data contribute important context: each Kit showed optimal concentration ranges that balanced encouraging effector cell activation and effective blast lysis. These advantageous concentration corridors of PGE_1_, PGE_2_, and OK-432 can now be tested *in vivo* or in proof-of-concept experiments in rodents. Our data highlight important pitfalls for clinical translation as too low concentrations of response modifiers may be ineffective, while too high concentrations may potentially be toxic (‘goldilocks’ principle) (42). It remains to be studied if blast lysis and clinical responses (or at least stabilization of the disease) can be achieved in off-label trials in patients with relapsed/refractory AML that are out of other treatment options. In general, our findings would argue for ramp-up dosing schedules that start at the lowest response modifier concentrations that were effective in generating DC_leu_ while maintaining efficient blast lysis (e.g., 0.5 µg/mL for PGE_1_, 5 µg/mL for OK-432, 0.5 µg/mL for PGE_2_). Because current literature remains limited in regard to the precise translation of *ex vivo* to physiologic conditions, such strategies that ‘start low and go slow’ appear prudent.

## Conclusions

In summary, our *ex vivo* data show that varying the concentrations of response modifiers within immunomodulatory Kits (M, I, K) influences their capacity to generate (mature) DC/DC_leu_. Using Kit-pretreated leukemic WB samples to stimulate immune cells following MLC increases blast lysis compared to controls. Modulating the concentrations of PGE_1_ in Kit-M (‘medium’ concentration range) and OK-432 in Kit-I (‘high’ concentration range), we were able to increase (mature) DC_leu_ generation, activate T effector and memory cells (after MLC), and improve blast lysis. Finally, correlation analyses revealed positive correlations of DC subtypes with T effector T_EM_/T_CM_ cells and improved blast lysis especially for patient samples pretreated with ‘medium’ (standard) concentrations of PGE_1_.

## Data Availability

The original contributions presented in the study are included in the article/**Supplementary Material**. Further inquiries can be directed to the corresponding author.
