# Supplementary material for: Concentration-dependent effects of immunomodulatory cocktails on the generation of leukemia-derived dendritic cells, DCleu mediated T-cell activation and on-target/off-tumor toxicity
Source: Front Immunol. 2025 Jan 30;15:1527961. doi: 10.3389/fimmu.2024.1527961 (PMC11821930; doi:10.3389/fimmu.2024.1527961)
Supplement: Supplementary file 1 [file DataSheet1.pdf]

Supplemental Material

Supplemental Methods

Breakdown of cell types in patient samples

Flow cytometric analysis of peripheral blood samples revealed the following average distribution of cell subtypes: blasts 37.1% (range 7–82%), B-cells 2.8% (0.66–8.42%), T-cells 9.4% (1.14–23.34%), NK-cells 4.5% (0.56–9.05%), CIK-cells 2.5% (0.29–4.35%), and monocytes 5.4% (1.01–17.99%).

Cell staining for flow cytometry

Cells were stained with various monoclonal antibodies (moAbs) conjugated with fluorescein isothiocyanate (FITC), phycoerythrin (PE), phycoerythrin-cyanine7 tandem-conjugate (PE- Cy7) or allophycocyanin (APC). Antibodies were provided by Beckman Coulter[a] (Krefeld, Germany), Becton Dickinson[b] (Heidelberg, Germany), Miltenyi Biotec[c] (Bergisch Gladbach, Germany), BioLegend[d] (Koblenz, Germany) and Santa Cruz Biotechnology[e] (Heidelberg, Germany). For analyses FITC-conjugated moAbs CD3[b], CD14[a], CD15[a], CD25[a], CD33[a], CD34[a], CD45RO[a], CD65[a], CD71[a]; PE-conjugated moAbs CD3[a], CD4[b], CD34[a], CD56[a], CD65[c], CD80[a], CD83[a], CD117[a], CD127[a] and CD206[a]; PE-Cy7-conjugated moAbs CD3[a], CD4[a], CD14[b], CD15[b], CD19[a], CD33[a], CD34[a], CD56[a], CD65[c], CD117[a], CD197[b]; APC-conjugated moAbs CD3[a], CD4[b], CD14[a], CD15[b], CD19[a], CD34[a], CD56[a], CD65[c], CD69[b], CD83[b], CD117[a], CD206[b], CD209[b] were used. Non-viable cells were detected with 7AAD[b].

Supplemental Figures

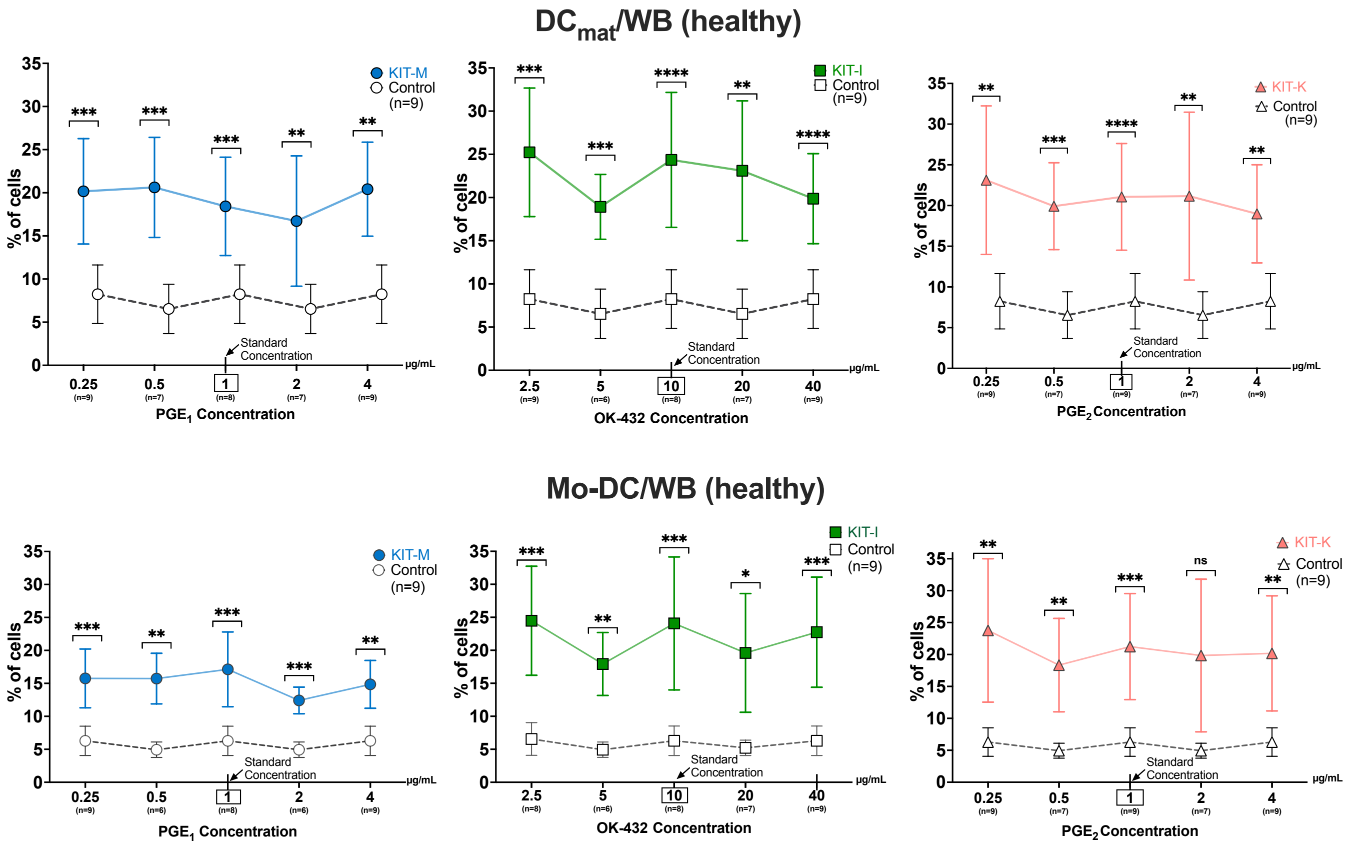

Supplemental Figure 1. DC_{mat} and Mo-DC generation using Kits with fixed standard concentrations of GM-CSF (800 U/mL) and varying concentrations of PGE_1, OK-432 and PGE_2 (from left to right). A box and arrow indicate the respective standard concentration**.** Data are presented as mean and 95% confidence intervals. Bonferroni's and Tukey's multiple comparison test were performed to calculate statistics, ****p <0.001, ***p<0.01, **p<0.05, *p<0.1 borderline significant, p>0.1 not significant (ns).

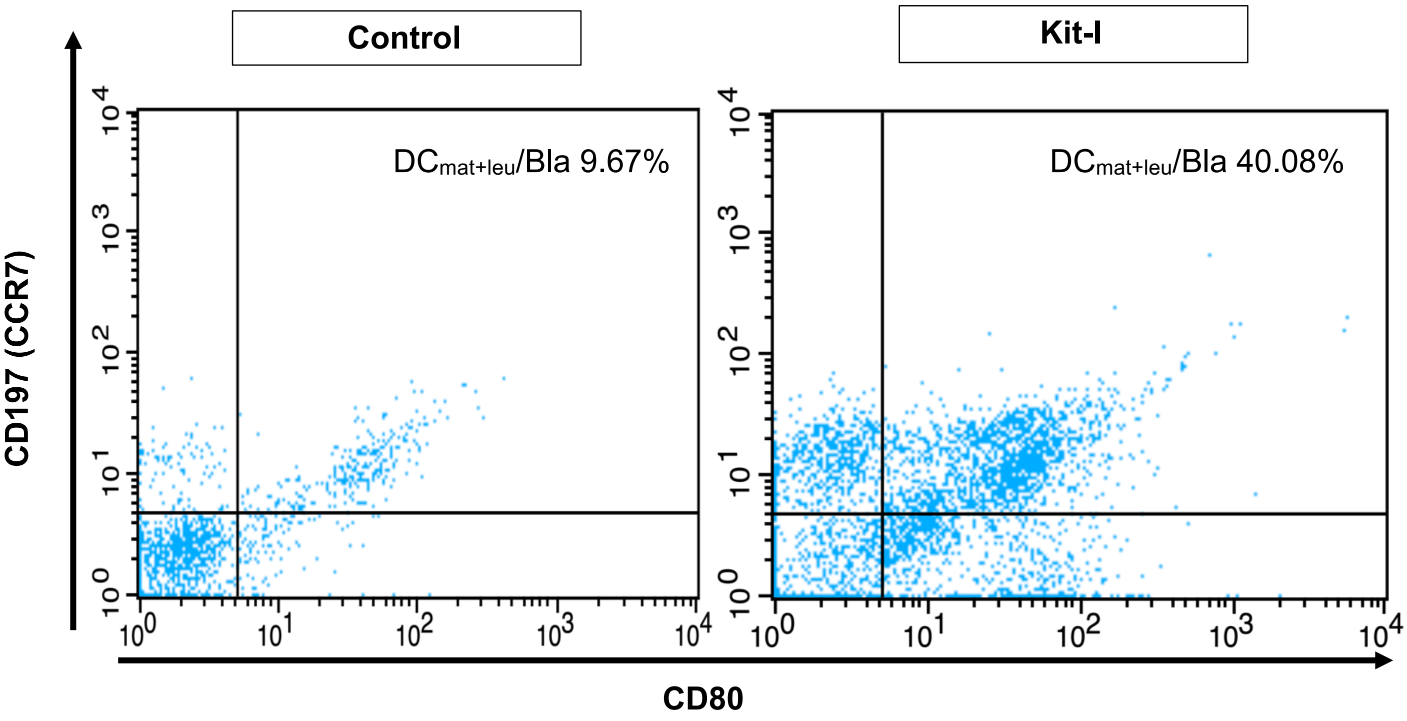

Supplemental Figure 2. Flow cytometric analyses of gated blasts show coexpression mature, leukemia-derived DCs with control and Kit-I. Exemplary plots show $DC_{mat+leu}$, characterized by the coexpression of the blast marker, the DC marker and CCR7.

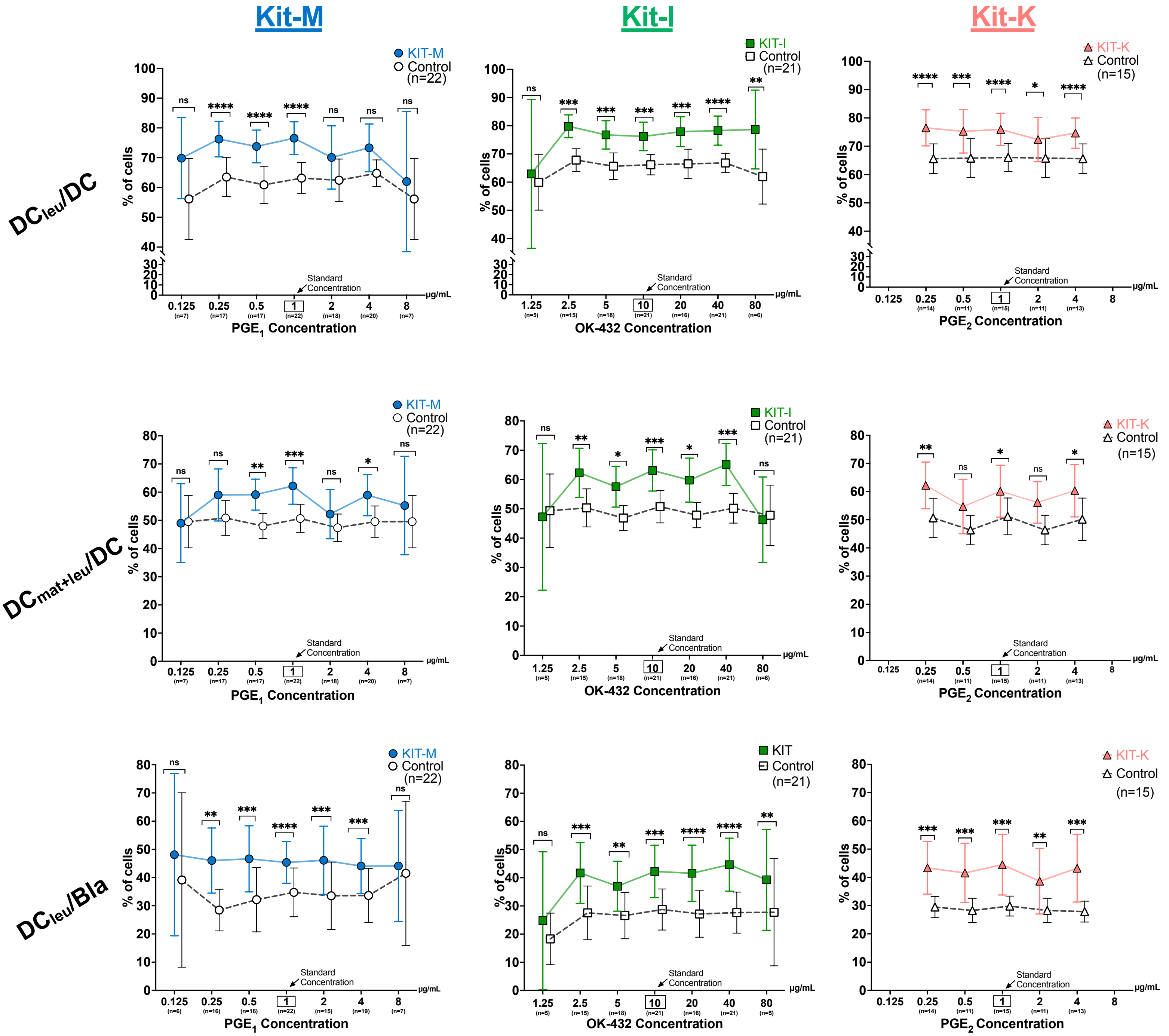

Supplemental Figure 3. Frequencies of DC_{leu}/DC, $DC_{mat+leu}/DC$ and DC_{leu}/Bla for KIT-M (●), KIT-I (■), KIT-K (▲) and control using fixed concentrations of GM-CSF (800 U/mL) and varying concentrations of PGE_1, OK-432 and PGE_2. Data are presented as mean ± 95% confidence intervals. Bonferroni's and Tukey's multiple comparisons tests were performed to calculate statistics, p-values are shown above the line graphs, ****p <0.001, ***p<0.01, **p<0.05, *p<0.1 borderline significant, p>0.1 not significant (ns). Abbreviations of cell subtypes are given in **Table 2**.

Representative flow cytometry scatter plots of CD8+$T_{naïve}$, CD8+T_{EM} and CD8+T_{CM}.

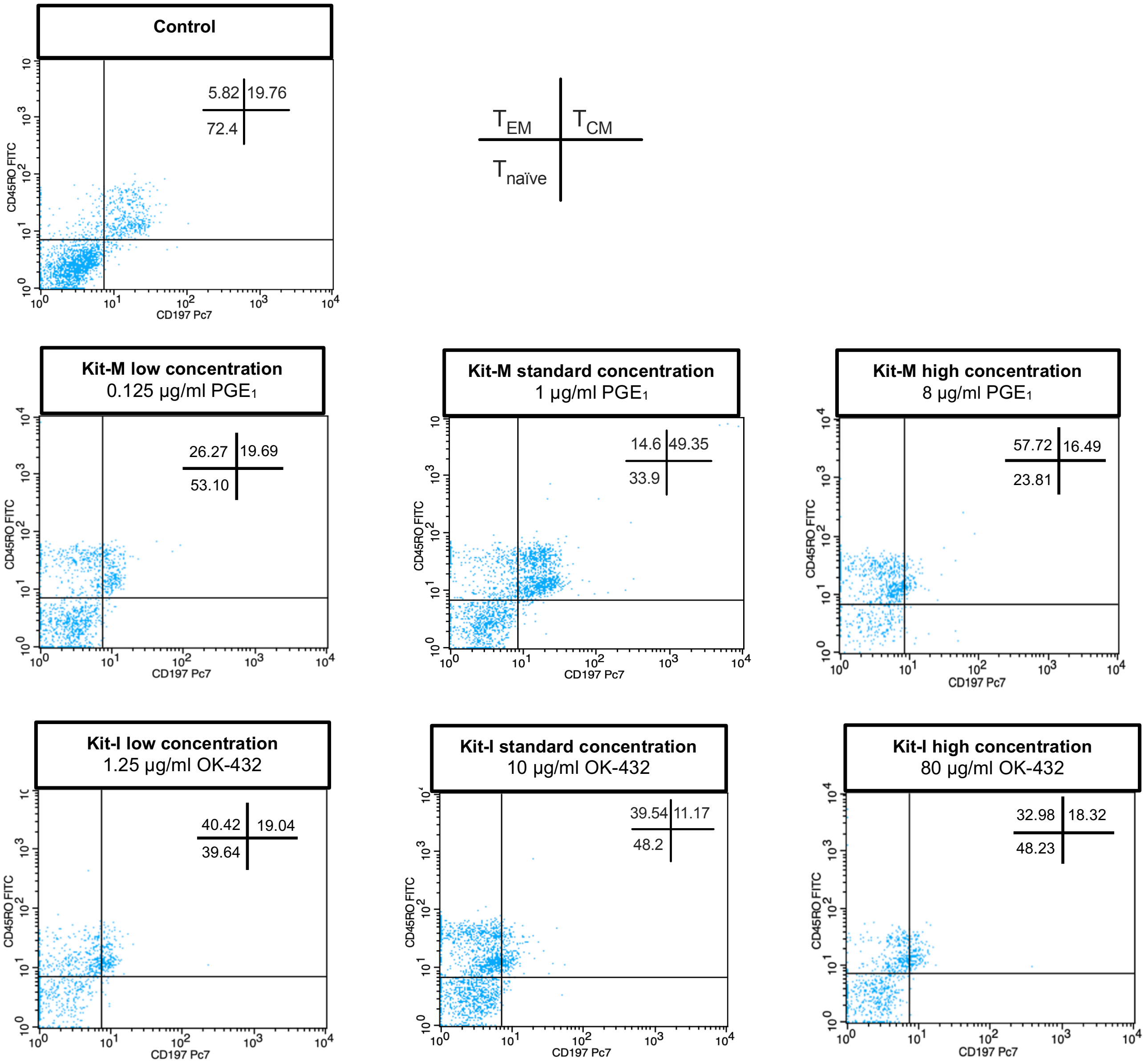

Supplemental Figure 4. Exemplary scatter plots show the distribution of T_{EM} and T_{CM} cells among CD3+CD8+ T-cells in MLC, which was evaluated based on the expression of CD45RO+ and CD197.

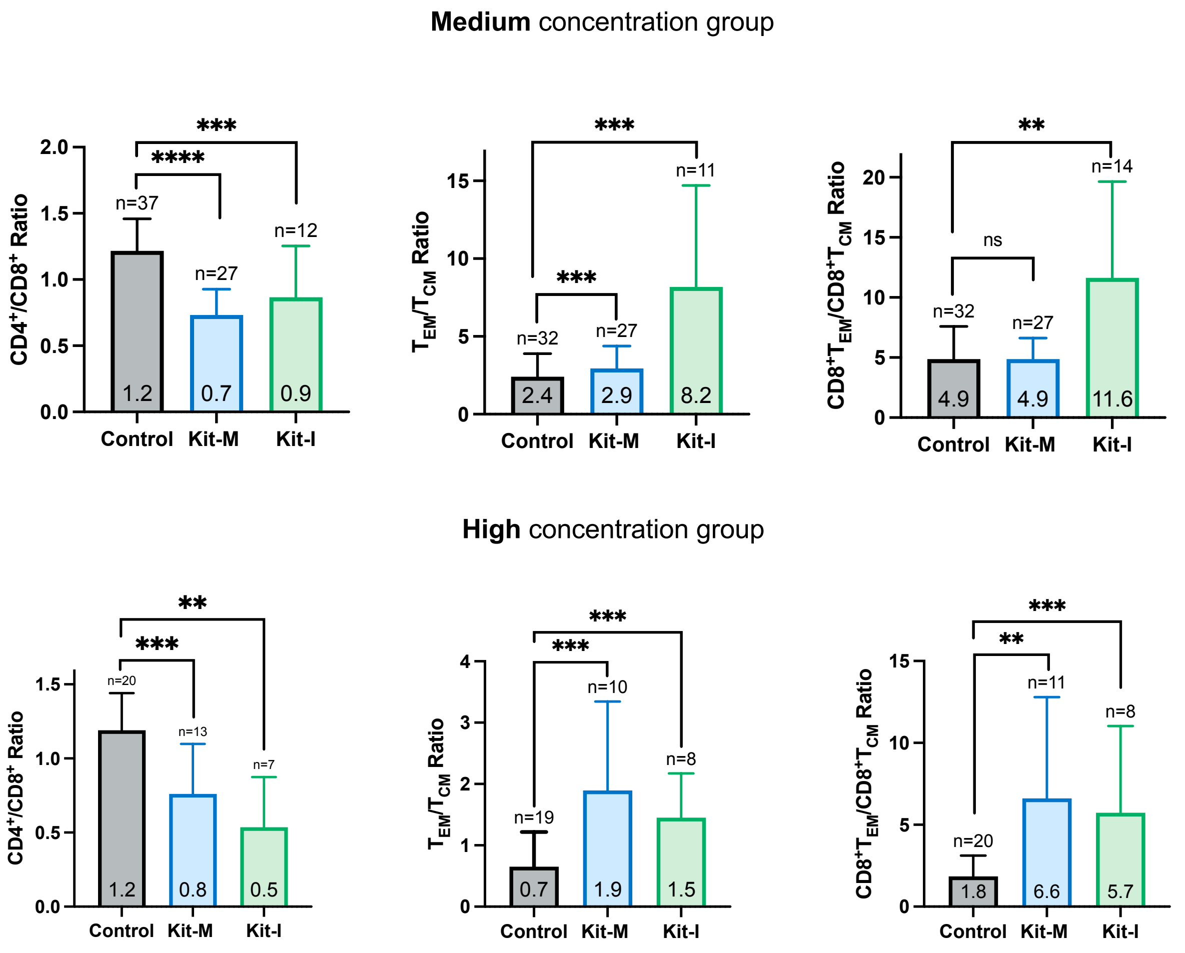

Supplemental Figure 5. CD4+:CD8+, T_{EM}:T_{CM} and CD8+T_{EM}:CD8+T_{CM} ratios in MLC after T-cell enriched leukemic WB in Kit-treated vs untreated (control) samples. **A)** Medium concentration group of Kit-M (0.5, 1 and 2 µg/mL PGE_1) and Kit-I (5 and 10 µg/mL OK-432) vs control **B)** High concentration group of Kit-M (4 and 8 µg/mL PGE_1) and Kit-I (20, 40 and 80 µg/mL OK-432) vs control. Significance was determined by Wilcoxon matched paired signed rank test, ****$p < 0.001$, ***$p < 0.01$, **$p < 0.05$, *$p < 0.1$ borderline significant, $p > 0.1$ not significant (ns).
